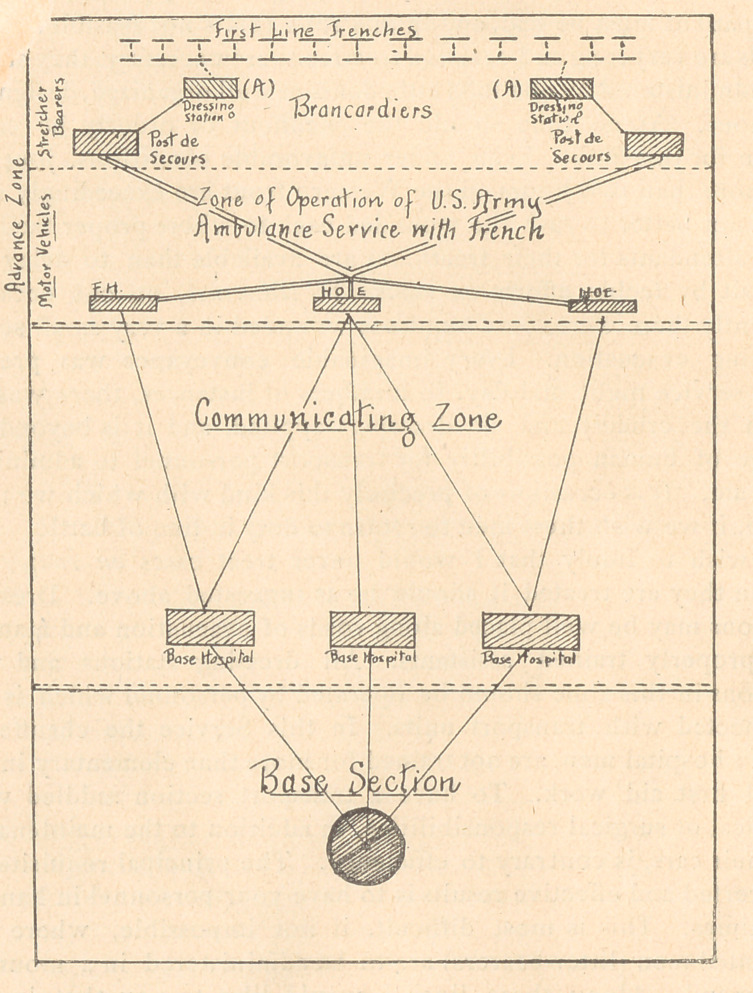# The Evacuation of Wounded by Motor Vehicles

**Published:** 1918-09

**Authors:** Percy L. Jones


**The Evacuation of Wounded by Motor Vehicles in the Rear Section of the Advance Zone.** Colonel PERCY L. JONES, M. C., N. A. The following is a detailed outline of the address:

*Evacuation of Wounded a Very Important Problem.*

The evacuation of wounded is a very important and very serious problem. No system will meet the needs under all conditions, as every evacuation on an unusually large scale must of necessity be a rule unto itself. Conveyances of all sorts should be as nearly as possible standardized, and where the quantity is not sufficient to meet the needs under stress, arrangements may also be made using empty vehicles pertaining to other arms of the service en route for replenishments. Even with this assistance, emergencies have arisen, and are constantly arising, where the quantity of transportation is woefully insufficient. All such details should be foreseen and arranged for from information obtained at Headquarters previous to emergency evacuation.

*Time Element of Chief Consideration.*

There are many factors to be considered in the evacuation of wounded, all of which are very important. They should be considered, however, with a view to shortening the period of non-effectiveness. This resolves itself into a time measure. Where the wounded occur in large numbers, it is frequently impossible for regimental medical personnel to administer the antitetanic serum. Other important factors are comfort, character of vehicles, distances, weather conditions, roads, etc. I think that it is generally conceded that the most important of the above measures is time; consequently, the efficiency of evacuation should be judged by this. This, however, applies more especially to wounds. There is no such thing as a non-transportable case in this zone.

*Subject of Evacuation of Wounded Best Treated By Zones.*

The subject as a whole may be well considered in zones, and each of these zones differs radically in the method of transportation, and in the care of patients in transit. The first zone is the most advanced section of the advance zone, where the transportation of wounded is accomplished by litter bearers. The second is just in the rear of that section, and comprises the remainder of the advance zone. The transportation of wounded in this zone is accomplished by motor vehicles, preferably the small Ford ambulances. This zone begins at the regimental dressing stations and ends at the H. O. E.'s, and should be considereded *solely* as a transport problem. The next zone is the communicating zone, extending from the H. O. E.'s in the French Service, casualty clearing stations in the British Service and evacuating hospitals in the American Service, to the base hospitals. This zone possesses ample facilities for transportation other than motor, such as railroad trains, trolly cars, water ways. etc. And, finally, the base zone, which needs no discussion in this paper.

*The Evacuation of Wounded in the Rear Section of the Advance Zone. The Sphere of Operations of U. S. Army Ambulance Service with the French Army.*

That portion of the advance zone behind the regimental aid stations back to the evacuation hospital, is the one which I am mostly interested in, and of which I am qualified to speak. I[illegible] zone, to my mind, the actual transportation of the patient [illegible] be completely divorced from any treatment *en route.* Thi[illegible] spur of the moment would seem inhumane, but the fact [illegible] that delay may mean comfort for one and infection for one [illegible] I believe this is the most important link in the entir[illegible] evacuation, for it is here that the time factor enters [illegible] it is here that contact must be maintained with u[illegible] The sanitary section should operate as a part of the di[illegible] should at no time be removed from the division to w[illegible] attached. To administer first aid during transit, proper [illegible] the patient must be had, and in this zone this is not feasible. [illegible] hauls are very long and care and treatment are necessary, they s[illegible] be administered by qualified attendants at well organized, design[illegible] stations. At no time should the chauffeur or orderly be cal[illegible] upon for this duty. Even under unfavorable conditions, journey[illegible] of more than two hours on the Western front are exceedingly rare. Is it not better to rush the wounded back to where proper facilities and attendants for their treatment are available than to delay the transit by feeble efforts *en route*? To illustrate, on one occasion recently there were some hundreds wounded in a very small sector waiting evacuation. Every imaginable conveyance was pressed into service night and day. In numbers of instances, there was not even the crudest first aid dressings applied, and it is beyond the range of human possibility for transport personnel to administer first aid. It is occasions of precisely this kind with which we must cope, if we wish these men to return to duty in line of battle. I do not wish to imply that I would never treat cases *en route*, but when they are treated it should be as suggested above. Dressing stations may be well placed along roads of evacuation and manned by properly trained assistants. All dressing stations and rest stations in this zone should be operated by personnel which is not connected with transport units. In this Service the chauffeurs, while hospital men, are not trained for more than elementary individual first aid work. To have a transport section saddled with medical or surgical responsibilities, in addition to the maintenance of their cars, is contrary to efficiency. The principal requisite for concerted and effective results is to have your personnel in hand at all times. This is most difficult, if not impossible, where dismounted men (litter bearers) are to be administered in a mounted command. Along these lines I would like to say that in my opinion I believe it very unwise to have mixed types of vehicles in an ambulance section. Each section, or company, should have one f car, some of the principal reasons being to obviate the v of having multiple sets of spare parts, multiple sets of ols, mechanics versed in more than one type of machine, sity of having to depend upon separate parts for supplies enance, etc.

*d Ambulance Best Adapted to Advance Zone Work.*

a doubt the best car for the advance work is the Ford ; small, light, easy to run, easy to maintain, simply constructed, economical in the consumption of gas, does not take much road space, and can be handled by one man when necessary.

What it lacks in capacity is more than compensated for by the above advantages.

Animal transportation should never be used in front line work, unless it is serving units which use only animal transportation. This on the Western front is very rare.

*Differentiating Features of the U. S. Army Ambulance Service.*

The American Automobile Sanitary Service closely resembles the British evacuation service in the advance zone. The U. S. Army Ambulance Service uses that of the French. In any system of evacuation in this zone, the fact must not be lost sight of that sufficient elasticity should be provided for in order to keep in constant touch with the units served. When avoidable, ambulances should never travel in convoys, as delays are very much increased thereby in passing troops, etc. Care should be used in selecting roads. Much harm can be done by delaying troops and munitions. Driving a motor ambulance in this service differs from the ordinary convoy work of supply services, where it is only necessary to follow a leader. In addition to a fair knowledge of map reading, the ability to orient onesself, and a knowledge of the conventional map signs, alertness, and individuality are required, which are not essential in ordinary work. These and other numerous essential points should be known by the chauffeur before being allowed to operate in the advance zone. This Service has its own school for that purpose. All replacement troops for this Service arriving from the United States are given an intensive course in the duties of this zone.

## Figures and Tables

**Figure f1:**
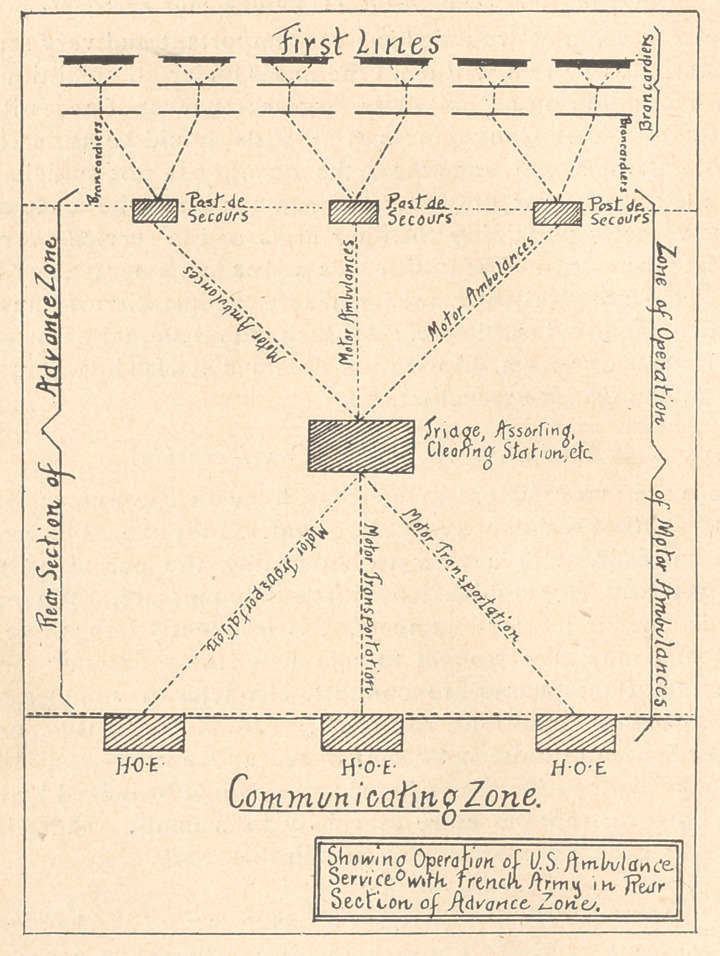


**Figure f2:**